# A genomic region involved in the formation of adhesin fibers in *Bacillus cereus* biofilms

**DOI:** 10.3389/fmicb.2014.00745

**Published:** 2015-01-13

**Authors:** Joaquín Caro-Astorga, Alejandro Pérez-García, Antonio de Vicente, Diego Romero

**Affiliations:** Departamento de Microbiología, Facultad de Ciencias, Instituto de Hortofruticultura Subtropical y Mediterránea “La Mayora” (IHSM-UMA-CSIC), Universidad de MálagaMálaga, Spain

**Keywords:** *Bacillus cereus*, biofilm formation, amyloid, extracellular matrix, food-borne pathogens

## Abstract

*Bacillus cereus* is a bacterial pathogen that is responsible for many recurrent disease outbreaks due to food contamination. Spores and biofilms are considered the most important reservoirs of *B. cereus* in contaminated fresh vegetables and fruits. Biofilms are bacterial communities that are difficult to eradicate from biotic and abiotic surfaces because of their stable and extremely strong extracellular matrix. These extracellular matrixes contain exopolysaccharides, proteins, extracellular DNA, and other minor components. Although *B. cereus* can form biofilms, the bacterial features governing assembly of the protective extracellular matrix are not known. Using the well-studied bacterium *B. subtilis* as a model, we identified two genomic loci in *B. cereus*, which encodes two orthologs of the amyloid-like protein TasA of *B. subtilis* and a SipW signal peptidase. Deletion of this genomic region in *B. cereus* inhibited biofilm assembly; notably, mutation of the putative signal peptidase SipW caused the same phenotype. However, mutations in *tasA* or *calY* did not completely prevent biofilm formation; strains that were mutated for either of these genes formed phenotypically different surface attached biofilms. Electron microscopy studies revealed that TasA polymerizes to form long and abundant fibers on cell surfaces, whereas CalY does not aggregate similarly. Heterologous expression of this amyloid-like cassette in a *B. subtilis* strain lacking the factors required for the assembly of TasA amyloid-like fibers revealed (i) the involvement of this *B. cereus* genomic region in formation of the air-liquid interphase pellicles and (ii) the intrinsic ability of TasA to form fibers similar to the amyloid-like fibers produced by its *B. subtilis* ortholog.

## Introduction

A major food-safety problem in developing countries is the contamination of fresh, stored and packaged food by bacteria that decrease the shelf life of the product and cause human poisoning (Burnett and Beuchat, 2001). Consumption of raw vegetables and fruits, milk, eggs, mildly cooked rice or pasta is typically associated with the most common outbreaks of poisoning (Carlin et al., 2000; Kamga Wambo et al., 2011). The symptoms of food poisoning can be mild, such as vomiting and diarrhea, or more severe, such as bacteremia; in severe cases, it can cause death of the patients. Bacterial strains of *Escherichia coli*, *Salmonella*, *Enterococcus*, *Lysteria* and *Bacillus cereus* are recurrent etiological agents of poisoning outbreaks (Berger et al., 2010). Epidemiological studies of these outbreaks have revealed that product contamination occurs before the manufacturing step. Irrigation with wastewater and the use of natural plant strengtheners lead to contamination of vegetables by enteropathogenic *E. coli* and *Salmonella* strains (Berger et al., 2010).

Several *B. cereus* strains are commonly observed as the etiological agents of poisoning outbreaks, severe bacteremia and septicemia (Bottone, 2010). *B. cereus*, a naturally inhabitant of soils, is frequently isolated from fresh vegetables and ready-to-eat vegetable-based food and is implicated in outbreaks of gastrointestinal diseases, abdominal pains, and watery diarrhea (Elhariry, 2011). *B. cereus* causes two main types of poisoning: emetic and diarrheic. Emetic poisoning is associated with production of cerulide, a lipophilic toxin. This toxin is extremely heat stable, and it can be produced in food contaminated by *B. cereus* cells. Notably, cerulide may persist in the body for a long period, affecting different organs and eventually leading to patient death (Thorsen et al., 2011). Diarrheic poisoning is caused by another group of toxic molecules: enterotoxin Hemolysin BL (HBL), the non-hemolytic enterotoxin (NHE) and cytotoxin (CytK). However, the specific role of each toxin in symptom development has not been elucidated. NHE and CytK are individually sufficient to induce diarrhea; however, it is not known whether HBL acts similarly. Similar to cerulide, HBL, NHE, and CytK can be produced in food contaminated with *B. cereus* cells; however, the sensitivity of these toxins to low pH and digestive proteases prevents the development of diarrheic symptoms. Therefore, poisoning occurs due to enterotoxin production in the small intestine by *B. cereus* cells or spores that have been ingested (McKillip, 2000).

Colonization and persistence of *B. cereus* cells in fresh vegetables and fruits are required for intoxication. *B. cereus* produces spores highly resistant to stressful environments and are able to survive heat, dry conditions, sanitation procedures, and food-processing treatments; and also aggregates in bacterial communities called biofilms (Ball et al., 2008; Shaheen et al., 2010; Elhariry, 2011). Studies on the related bacterial species *B. subtilis* revealed that biofilms are natural reservoirs of spores and are as recalcitrant as spores to eradication therapies (Branda et al., 2005). Biofilm formation requires (i) a complex regulatory pathway that coordinates gene expression with external environmental conditions and (ii) structural components involved in the assembly of a protective extracellular matrix (Romero, 2013; Vlamakis et al., 2013). In *B. subtilis*, the extracellular matrix is composed of exopolysaccharides, the hydrophobin protein BlsA and the amyloid-like protein TasA (Branda et al., 2004; Romero et al., 2010; Kobayashi and Iwano, 2012; Hobley et al., 2013; Romero, 2013). Studies on bacterial ecology have focused on amyloid proteins because: (i) they retain the morphological and biochemical features of their pathogenic siblings in humans (Fowler et al., 2007), (ii) they are involved in multiple functions relevant to bacterial physiology and ecology (Chapman et al., 2002; Epstein and Chapman, 2008; De Jong et al., 2009; Dueholm et al., 2010; Romero et al., 2010; Schwartz et al., 2012), and (iii) they undergo a complex program leading to fibrillation (Blanco et al., 2012). In *B. subtilis* biofilms, TasA amyloid-like fibers constitute the protein skeleton that directs the assembly of the extracellular matrix (Romero et al., 2010).

Although biofilm formation has been studied in detail in *B. subtilis*, not much is known about this developmental program in *B. cereus*. Separate studies revealed that specific *B. cereus* elements are involved in biofilm formation: notably, *sinR* and *sinI*, *spo0A* or *abrB*, major regulators controlling the developmental program ending in biofilm formation of *B. subtilis* has also been demonstrated to play similar roles in biofilm formation of *B. cereus*. The SinR regulon in a strain of *B. thuringiensis* closely related to *B. cereus* contains the loci *sipW-tasA*, as it does in *B. subtilis*, but also the lipopeptide kurstakin, important for biofilm formation (Pflughoeft et al., 2011; Fagerlund et al., 2014). In addition, *B. cereus* appears to form wrinkly colonies and cell bundles in response to glycerol, manganese or milk, and this is proposed to be mediated by the kinase KinD as seen in *B. subtilis* (Shemesh and Chai, 2013; Pasvolsky et al., 2014). Other two major regulators of *B. cereus* with involvement in biofilm are PlcR, the main virulence regulator, and CodY, a repressor of branched aminoacids, which points toward the inevitable connection of virulence with biofilm formation in this bacteria species (Hsueh et al., 2006; Lindback et al., 2012). Besides this knolowledge on the biofilm-dedicated regulatory pathways, other studies have revealed the relevance on motility on adhesion to abiotic surfaces, or the presence of extracellular DNA and other uncharacterized proteins or polysaccharides in the extracellular matrix of biofilms of *B. cereus* (Auger et al., 2009; Vilain et al., 2009; Houry et al., 2010; Karunakaran and Biggs, 2011).

Because amyloid-like fibers are important for biofilm formation in diverse bacterial species, we examined the role of a genomic region encoding two orthologs, TasA and CalY, of the *B. subtilis* TasA amyloid-like protein in biofilm formation by *B. cereus*. Using mutagenesis analysis, we revealed that this region is important for biofilm assembly in *B. cereus* CECT148. Electron microscopy analysis revealed the presence of TasA fibers on the *B. cereus* cell surfaces, similar to those formed by *B. subtilis* TasA. Furthermore, by heterologous expression of *B. cereus* alleles in *B. subtilis* mutants lacking different components required for amyloid-like fiber assembly, we observed that *B. cereus* TasA functions similar to the endogenous *B. subtilis* TasA protein: (i) it is involved in the formation of wrinkles in the air-liquid interphase pellicle, a visual feature of mature biofilms, (ii) the pellicles are positively stained with the amyloid-specific dye Congo Red and (iii) abundant and robust fibers are assembled on cell surfaces.

## Materials and methods

### Bacterial strains and culture conditions

The bacteria used in this study are listed in Table 1. Bacteria were routinely grown in Ty broth (1% tryptone, OXOID), 0.5% yeast extract (OXOID), 0.5% NaCl, 10 mM MgSO_4_, and 1 mM MnSO_4_. Biofilm assays were performed either in TY or MSgg broth (100 mM morpholinopropanesulfonic acid (MOPS) (pH 7), 0.5% glycerol, 0.5% glutamate, 5 mM potassium phosphate (pH 7), 50 μg/ml tryptophan, 50 μg/ml phenylalanine, 2 mM MgCl_2_, 700 μM CaCl_2_, 50 μM FeCl_3_, 50 μM MnCl_2_, 2 μM thiamine, and 1 μM ZnCl_2_) (Branda et al., 2001). Antibiotics were used when required at the following concentrations (final): MLS, 1 μg/ml erythromycin, 25 μg/ml lincomycin, spectinomycin 100 μg/ml, chloramphenicol 5 μg/ml, and kanamycin 10 μg/ml.

**Table 1:** **Strains used in this study**.

**Strain**	**Derivative strain**	**Genotype**	**Reference**
*B. subtilis* 168		Prototroph	Branda et al., 2001
*B. subtilis* NCIB3610		Undomesticated prototroph	Branda et al., 2001
*B. subtilis* 168	SSB149	*(tapA-sipW-tasA)::spc*	Branda et al., 2004
*B. subtilis* NCIB3610		*(tapA-sipW-tasA)::spc*	Branda et al., 2004
*B. subtilis* NCIB3610	FC268	*(tapA-sipW-tasA)::spc, amyE::(tapA::cm-sipW-tasA)::spc*	Chu et al., 2006
*B. subtilis* NCIB3610	CA017	*tasA::km*	Romero et al., 2010
*B. subtilis* NCIB3610	JCA32	*tasA::km, lacA::P_hyperspank_-sipW-tasA-bc_1280-calY-mls*	This study
*B. subtilis* NCIB3610	DR6	*tasA::km, lacA::P_hyperspank_-tasA-mls*	Romero et al., 2011
*B. subtilis* NCIB3610	JCA33	*(tapA-sipW-tasA)::spc, amyE::((tapA::cm)-sipW-tasA), lacA::P_hyperspank_-sipW-tasA-bc_1280-calY-mls*	This study
*B. subtilis* NCIB3610	JCA34	*(tapA-sipW-tasA)::spc, lacA::P_hyperspank_-sipW-tasA-bc_1280-calY-mls*	This study
*B. subtilis* NCIB3610	JCA35	*tasA::km, amyE::P_hyperspank_-calY-spc*	This study
*B. subtilis* NCIB3610	JCA36	*tasA::km, amyE::P_hyperspank_-sipW-tasA-spc*	This study
*B. subtilis* NCIB3610	JCA56	*(tapA-sipW-tasA)::spc, lacA::P_hyperspank_ sipW-calY-mls*	This study
*B. subtilis* NCIB3610	JCA57	*(tapA-sipW-tasA)::spc, amyE::(tapA::cm-sipW-tasA), lacA::P_hyperspank_-sipW-calY-mls*	This study
*B. subtilis* NCIB3610	JCA58	*tasA::km, lacA::P_hyperspank_-sipW-calY-mls*	This study
*B. subtilis* NCIB3610	JCA90	*(tapA-sipW-tasA)::spc, amyE:: P_hyperspank_-sipW-tasA-spc*	This study
*B. subtilis* NCIB3610	JCA91	*(tapA-sipW-tasA)::spc, amyE::((tapA::cm)-sipW-tasA), lacA::P_hyperspank_-sipW-tasA-mls*	This study
*B. subtilis* NCIB3610	JCA92	*tasA::Km, amyE::P_hyperspank-_tasA-spc*	This study
*B. cereus* CECT148		Type strain	Spanish Colection of Type Strains
*B. cereus* CECT148	JCA110	*sipW::Mls*	This study
*B. cereus* CECT148	JCA111	*tasA::Km*	This study
*B. cereus* CECT148	JCA113	*calY::Mls*	This study
*B. cereus* CECT148	JCA114	*(sipW-tasA-bc_1280-calY)::Mls*	This study

### RNA purification and RT-PCR

A 3 ml culture of *B. cerues* CECT148 in LB was growth without agitation at 30°C. After 24 h, the tube was vortexed to resuspend the ring of biomass adhered to the wells of the tube and centrifuged at 7000 g 1 min to collect cells. The cells were washed and lysed in 1 ml BirnBoim A solution (10% sucrose; 10 mM TrisHCl, pH8.1; 10 mM EDTA; 50 mM NaCl), supplemented with 20 μg/ml lysozyme from chicken egg white (Sigma) for 30 min 37°C, and eventually sonicated (power discharge 0.5 s, pause 0.5 s, amplitude 20% and 20 pulses). Cells were pelleted at 7000 g 1 min, and the pellet resuspended in 1 ml of Trizol (Trireagent, Trisure) with 10 μ l of proteinase K, and incubated at 60°C 20 min. After that, 200 μ l of chloroform were added to the sample, mixed inverting the tube several times and centrifuged. Supernatant over the interface containing nucleic acids were collected carefully without disrupting the interphase. The subsequent steps for purification of RNA was performed using a commercial kit (Nucleospin RNA Plant, Macherey-Nagel). The integrity of the RNA extraction was tested by electrophoresis in agarose gel and cDNA was obtained using Titan One RT-PCR System (Roche). To prove which genes constitute an operon we performed PCR with cDNA as template using primers between genes to test which are transcribed in the same RNA molecule; and primers inside each gene to test if they were expressed. Genomic DNA was purified using the commercial kit *UltraClean Microbial DNA Isolation* of *MOBIO* Laboratories. Positive controls for each primer pair were included using genomic DNA as template and for negative controls RNA extraction as template to ensure that RNA extraction was not contaminated with genomic DNA. (Specific primers are specified in Table S1).

### Construction of *B. cereus* mutants

*B. cereus* mutants were obtained by electroporation using derivatives of the plasmid pMAD (Arnaud et al., 2004). Primers used to generate the mutagenesis constructs are listed in Table S1. The constructs were created by joining PCR, as previously described (López et al., 2009). In the first step, regions flanking the target genes and antibiotic-resistance cassettes were amplified separately, purified, and used for the joining PCRs. These PCR products were digested with enzymes BamHI and NcoI and cloned into the pMAD vector digested with the same enzymes (Arnaud et al., 2004). The resulting suicide plasmids were used to transform *B. cereus* electrocompetent cells as described previously (Pflughoeft et al., 2011). Electroporations were performed with 4 μ g of plasmids and 100 μ L of electrocompetent *B. cereus* in 0.2-mm cuvettes using the following electroporation parameters: voltage 2500 kV, capacitance 25 μ F, resistance 350 Ω. The electropored cells were seeded in LB plates supplemented with X-Gal and erythromycin for 72 h at 30°C. Blue colonies were selected and restreaked as previously described to trigger allele replacement (Arnaud et al., 2004). Finally, white colonies that were sensitive to MLS were selected, and deletion of the target gene was verified by colony PCR analysis and sequencing of the amplicons.

### Heterologous expression of *B. cereus* alleles in *B. subtilis* mutants

*B. cereus* alleles were amplified with specific primers (Table S1), digested and cloned into the integrative plasmid pDR111 (for ectopic integration at the *amyE* locus), digested with the same enzyme. When required, fragments containing the *P_hyperspank_*-promoter and the inserts were sub-cloned into the integrative plasmid pDR183 for ectopic integration at the *lacA* locus (López et al., 2009; Romero et al., 2011). The resulting integrative plasmids were used to transform *B. subtilis* 168 by natural competence; subsequently, using generalized transduction with Spp1 phages, the constructs were introduced into the recipient *B. subtilis* 3610 strains (Romero et al., 2011). The transformants were selected by antibiotic resistance and tested by PCR.

### Biofilm assays

*B. subtilis* biofilm formation was analyzed in MSgg medium (Branda et al., 2006). For pre-cultures, each strain was grown in LB agar with the required antibiotic at 37°C for 8 h. A colony was resuspended in 1 ml of MSgg, and 10 μl was used to inoculate 1 ml of MSgg in 24-well plates, and the plates were incubated without agitation at 30°C. The pellicles were examined for the presence of wrinkles, a morphological feature of mature *B. subtilis* biofilms (Branda et al., 2006).

*B. cereus* biofilm formation was monitored by testing bacterial adhesion to abiotic surfaces and staining with crystal violet (O'toole et al., 1999). Cultures were grown in TY at 30°C without agitation. One milliliter of a 1% solution of crystal violet in water was added to each well of a 24-well-plate. After 5 min of incubation, the plates were rinsed five times by immersion in tap water and were left inverted to dry on the bench for at least 45 min. The crystal violet was then resuspended with 50% acetic acid. The resuspended solution was diluted 1/10, and the absorbance was measured at 595 nm.

### Congo red assay

Staining of *B. subtilis* pellicles with the amyloid dye Congo Red was performed as described above but using Ty medium supplemented with Congo Red and Coomassie Brilliant Blue G at final concentrations of 20 and 10 μg/ml; the dyes were filtered and added to autoclaved Ty medium (Romero et al., 2010). The same procedures were used to stain *B. cereus* pellicles but grown in 4.5 cm diameter plates.

### Immunolabeling and transmission electron microscopy analysis

Each strain was grown in LB agar with the required antibiotic at 37°C for 8 h. A colony was resuspended in 1 ml of Ty (*B. cereus* strains) or MSgg (*B. subtilis* strains) and 10 μl was inoculated in 1 ml of Ty or MSgg in 24-well plates; the plates were incubated without agitation at 30°C for 24 h. Cooper Grids for TEM were deposited on the air-liquid interface and incubated overnight. The grids were contrasted using 1% uranyl acetate for 2 min, rinsed by submersion in distilled water 2 min twice and then dried prior to examination. For immunolabeling assay, samples were floated on blocking buffer (1% non-fat dry milk in PBS with 0.1% Tween20) for 30 min, on anti-TasA of *B. subtilis* 1:150 for 2 h, rinsed in PBST 30 min with a buffer change every 5 min, floated in goat-anti-rabbit 40 nm gold secondary antibody (TedPella) 1:50 at 37°C 1 h, rinsed in PBST and in ultrapure water four times for 5 min each. Samples were dried at RT and contrasted as previously described. Samples were visualized and photographed in a JEOL JEM-1400 transmission electron microscope.

## Results

### *B. cereus* encodes two orthologs of the *B. subtilis* amyloid-like protein TasA

**Figure 1:**
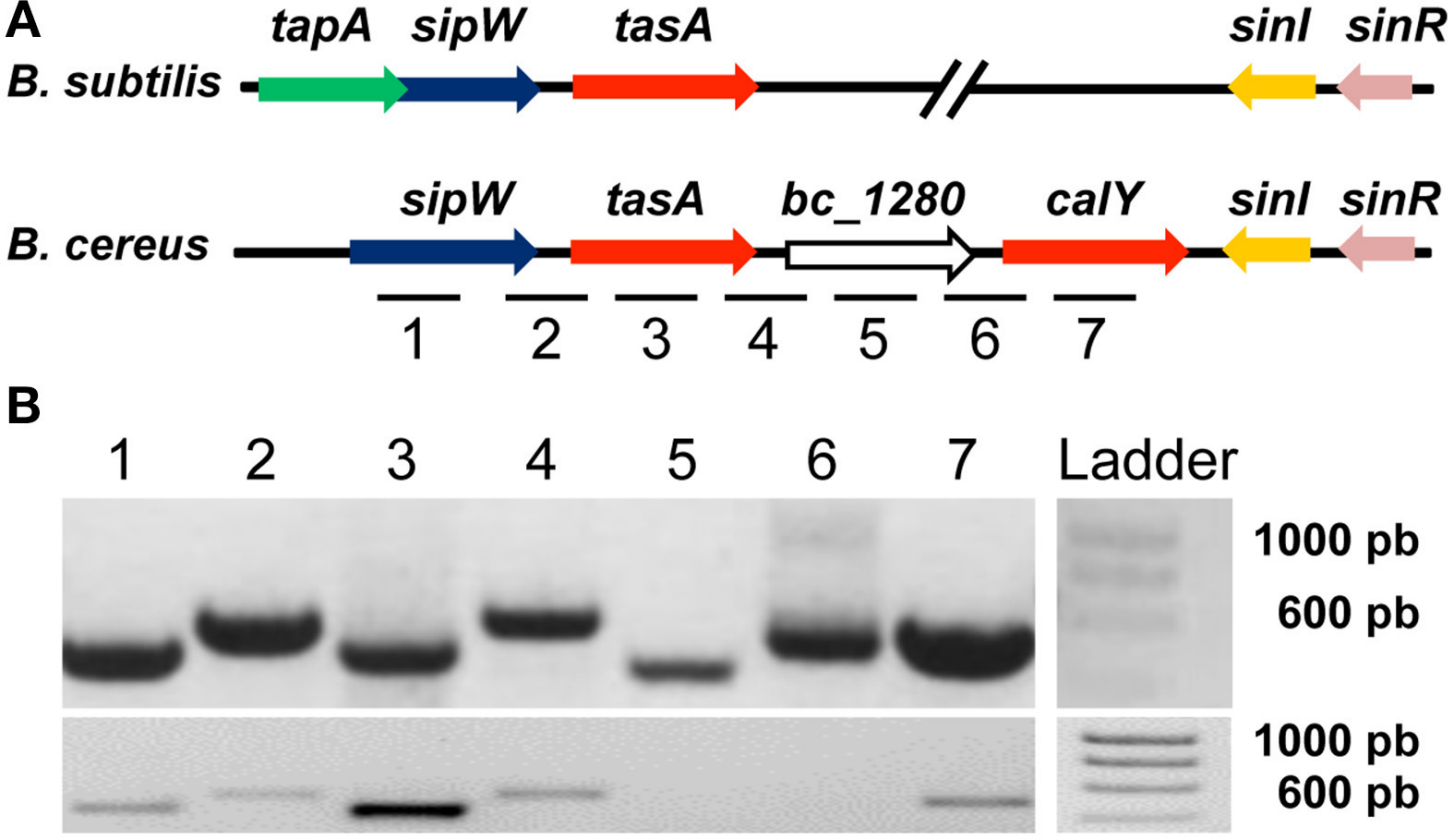
***Bacillus cereus* has genes orthologous to *B. subtilis* genes, which are required for the formation of amyloid-like fibers**. **(A)** Comparison of the *B. subtilis* and *B. cereus* genomic regions required for biofilm formation. The *tapA* operon (*tapA_op_*) is involved in the formation of TasA amyloid-like fibers; TapA (accessory protein for biofilm formation); SipW (signal peptidase that processes immature TapA and TasA); TasA (the major subunit of the amyloid-like fibers). SinR is a negative regulator of the *tapA_op_*, and SinI antagonizes SinR. The *B. cereus* genomic region contains genes orthologous to *tasA* (*tasA and calY*), *sipW*, *sinI* and *sinR* of *B. subtilis*, but lack *tapA*. **(B)** PCR on genomic DNA proved the functionality of the primers (top picture); RT-PCR analysis of RNA purified from a 24 h *B. cereus* culture reveals that *sipW* and *tasA* are co-transcribed but not *bc_1280* or *calY* (bottom picture). Lanes 1 to 7 are signals corresponding to the amplification of the inter or intragenic sequences marked with the same numbers in Figure 1A.

*B. subtilis* biofilms are mainly composed of exopolysaccharides and the protein TasA. TasA can polymerize to form fibers that are morphologically and biochemically similar to amyloid proteins (Romero et al., 2010). A specific chromosomal region in *B. cereus* is similar to that of *B. subtilis* implicated in biofilm formation; this region encodes an ortholog of *sipW* and two orthologs of *tasA* (*tasA* and *calY*). Using the genome sequence of the type strain *B. cereus* ATCC14579 as reference, we identified all the genes of this region on *B. cereus* CECT148 (Figure 1A). It is important to mention, that an additional putative ortholog (*bc_4868*) with 29% identity to TasA of *B. subtilis* can be identified in the genome of *B. cereus* ATCC14579. This gene is predicted to encode a putative protease, and localized in the genome close to other protease-encoding gene, but we did not include it in our analysis. In *B. subtilis*, the genes *tapA, sipW* and *tasA* constitute an operon (*tapA_op_*) and we proposed that this organization is conserved in *B. cereus*. To test this hypothesis, we extracted RNA from a 24-h culture of *B. cereus* and performed RT-PCR analysis using primers specific to each gene; we examined the expression of these genes to test whether they were transcribed together. First we confirmed the primers worked properly doing PCR on genomic DNA of *B. cereus* CECT148 (Figure 1B top picture). In the RT-PCR analysis we observed that *sipW* and *tasA* constitute an operon; the locus *bc_1280* was not expressed under our experimental conditions, and *calY* was expressed independently (Figure 1B bottom picture).

### SipW-tasA and CalY are involved in *B. cereus* biofilm formation

**Figure 2:**
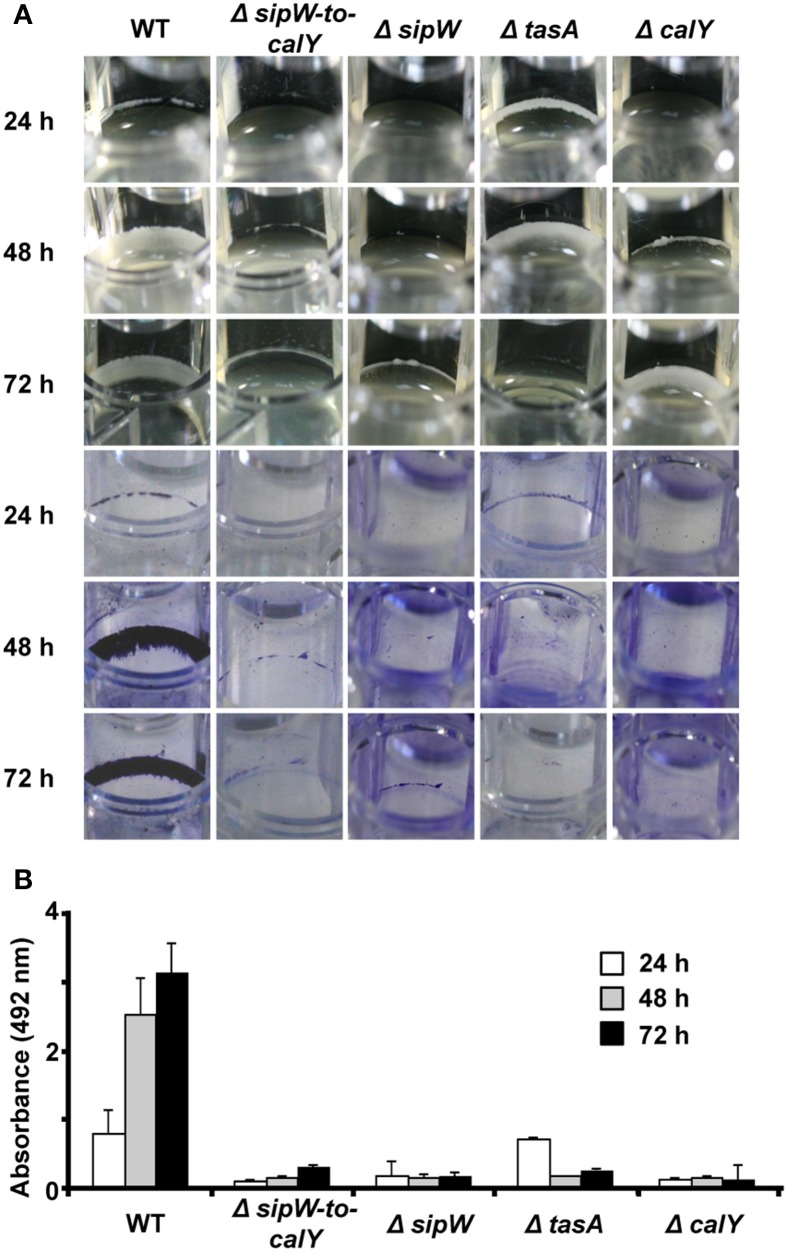
***sipW-tasA* and *calY* play complementary roles in *B. cereus* biofilm formation. (A)** Biofilms of *B. cereus* wild type and mutant cells were visualized as rings of biomass adhered to the wells in TY broth at 30°C without agitation (top pictures). The biomass-related rings were photographed after and before staining with crystal violet (bottom pictures). **(B)** Quantification of the amount of crystal violet retained in bacterial biomass. Data are the average and standard deviation from four independent experiments consisting of six internal replicates each.

Because *tapA_op_* is important for the assembly of biofilms in *B. subtilis*, we examined the role of *sipW-tasA* and *calY* in *B. cereus* biofilm formation. Biofilms were visualized by performing crystal violet staining on the biomass adhered to well surfaces (Figure 2). After 24 h of growth, wild-type cells formed visible rings, which grew in thickness up to 72 h (Figure 2A top pictures). At all these stages, the rings of adhered biomass were strongly stained with crystal violet (Figure 2A bottom pictures, and Figure 2B). Deletion of the *sipW-to-calY* region prevented the formation of similar biomass rings; a mutation in *sipW* caused the same phenotype. SipW is a signal peptidase involved in TasA processing, which facilitates efficient secretion of TasA from *B. subtilis* cells (Stover and Driks, 1999b); mutation of *sipW* eliminated biofilm formation ability. Notably, deletion of *tasA* conferred an unexpected phenotype: compared to the wild type strain, a thicker biomass ring was observed in the *tasA* deletion strain at 24 h, which disappeared at 72 h. Upon staining with crystal violet, the biomass was extruded from the wells at each stage. Deletion of *calY* caused a different phenotype: the biomass adhered to the wells was thicker after 72 h of growth; however, similar to the phenotype of the *tasA* mutant, the biomass did not bind tightly to the wells upon crystal violet staining. These observations revealed that this region is important for biofilm formation in *B. cereus* and that *tasA* and *calY* potentially participate at different stages of biofilm formation, including initial attachment and maturation.

### TasA forms more abundant fibers than CalY in *B. cereus* biofilms

**Figure 3:**
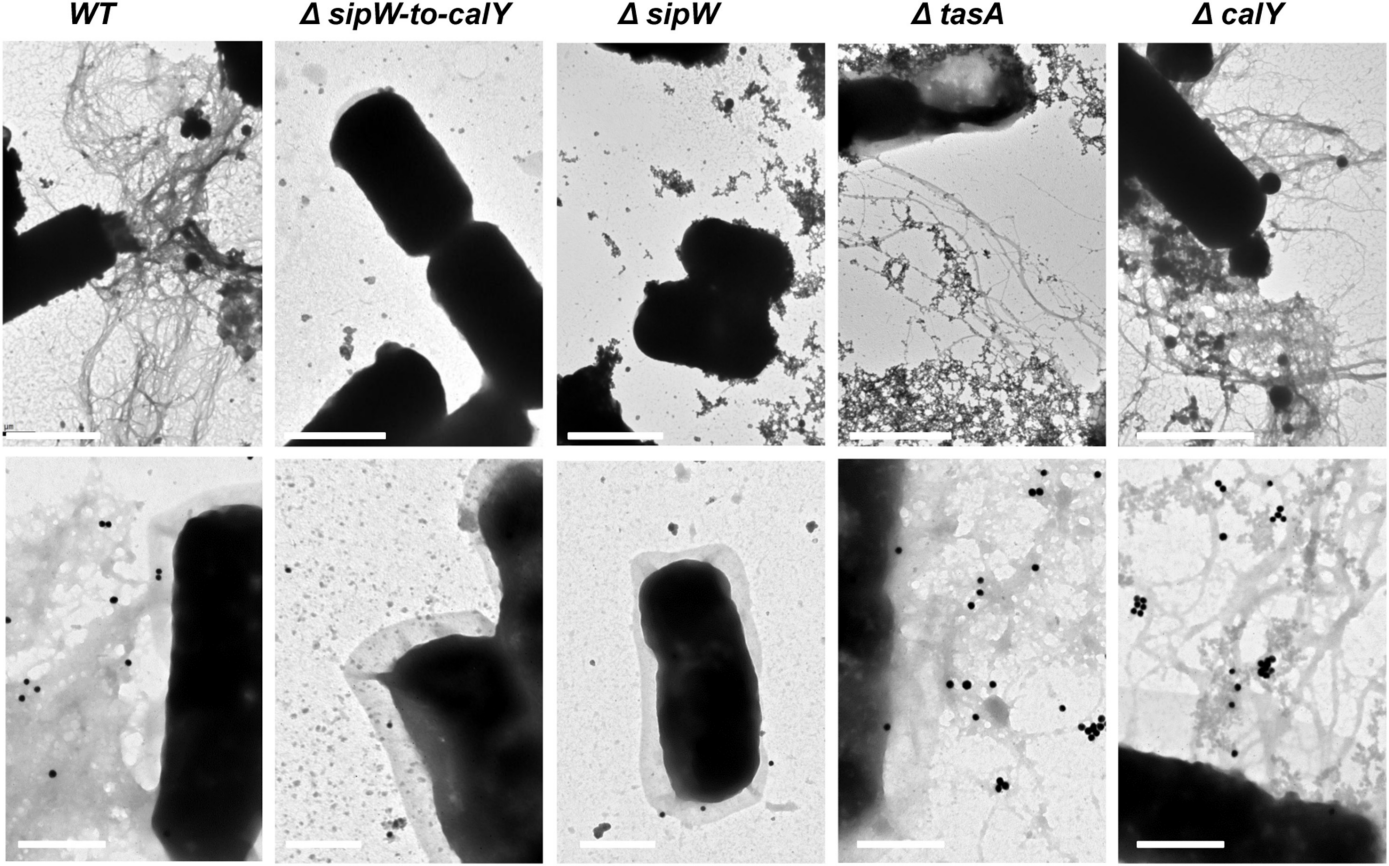
**TasA forms more abundant fibers than CalY in *B. cereus***. Biomass rings of *B. cereus* strains were isolated after 24 h of growth, contrasted with uranyl acetate and analyzed using transmission electron microscopy (top row), or immunolabeled with primary anti-TasA antibodies (1:150) and secondary antibody conjugated to 40 nm gold particles (1:50) before contrasting with uranyl acetate and visualization (bottom row). Bars equal 1 μm (top row images) or 0.5 μm (bottom row images).

The formation of amyloid-like fibers in *B. subtilis* requires the function of two proteins, TasA and the accessory protein TapA (Romero et al., 2014). Bioinformatics analysis revealed that at the amino acid level, TasA and CalY share only 31 and 32% identity to *B. subtilis tasA*, respectively, but share 61% identity with each other. Based on these observations and the absence of a TapA ortholog in *B. cereus*, we proposed that TasA and CalY do not form fibers. To test this hypothesis, we use transmission electron microscopy to analyze *B. cereus* biomass adhered to wells (Figure 3, top row). Contrary to our hypothesis, *B. cereus* cells appeared highly decorated with fibers; however, cells with a deletion of this genomic region (Δ*sipw-to-calY*) or a mutation in *sipW* (Δ*sipW*) did not form fibers. Consistent with our previous biofilm experiments (Figure 2), cells of the single Δ*tasA* mutant, which expressed *calY*, produced thin and less abundant fibrils compared to the wild type. However, Δ*calY* mutant cells, which expressed *tasA*, formed abundant fibers on their surfaces, similar to the wild type. To confirm that TasA or CalY formed fibers, we performed immunoelectron microscopy using anti-TasA antibodies raised against *B. subtilis* TasA (Figure 3, bottom row). The cross immunereaction of *B. cereus* TasA with anti-TasA antibodies of *B. subtilis* was previously demonstrated (Pflughoeft et al., 2011). The fibers observed in wild type, Δ*tasA* and Δ*calY B. cereus* cells immunoreacted with the anti-TasA antibodies; however, no signal was observed in Δ*sipW-to-calY* or Δ*sipW* mutant cells.

**Figure 4:**
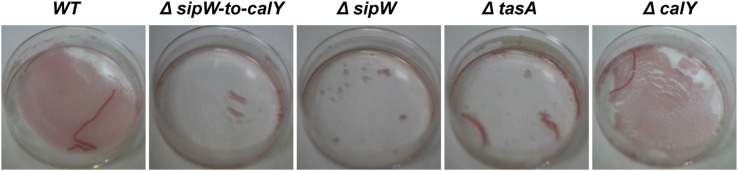
**Thin pellicles of *B. cereus* containing TasA bind the amyloid dye Congo Red**. Biofilms *of B. cereus* were grown in 4.5 cm diameter plates containing TY supplemented with a solution of Congo Red (20 μ g/mL) and Coomassie Brilliant Blue G (10 μ g/mL) for 5 days at 30°C. Top view pictures of pellicles after removing the spending medium.

Because *B. cereus* TasA forms fibers similar to *B. subtilis* TasA, we proposed that *B. cereus* biofilms would bind to amyloid dyes such as Congo Red. We performed biofilm experiments in 4.5-cm-diameter plates containing TY supplemented with Congo Red (Figure 4). Similar to the experiments performed with microtiter plates, no pellicles were observed during the initial stages of growth; however, after 5 days, a thin pellicle was visible in the *B. cereus* wild-type and Δ*calY* strains but not in Δ*sipW-to-calY*, Δ*sipW* or Δ*tasA* mutants. When the medium was removed, the remaining pellicle appeared stained with Congo Red. These observations suggested that (i) TasA fibers are more important than CalY fibers for producing pellicles in the air-liquid interphase and (ii) *B. cereus* TasA fibers stain similarly to TasA amyloid-like fibers of *B. subtilis*.

### Expression of the *B. cereus sipW-TO-calY* region rescues pellicle formation in *B. subtilis*

**Figure 5:**
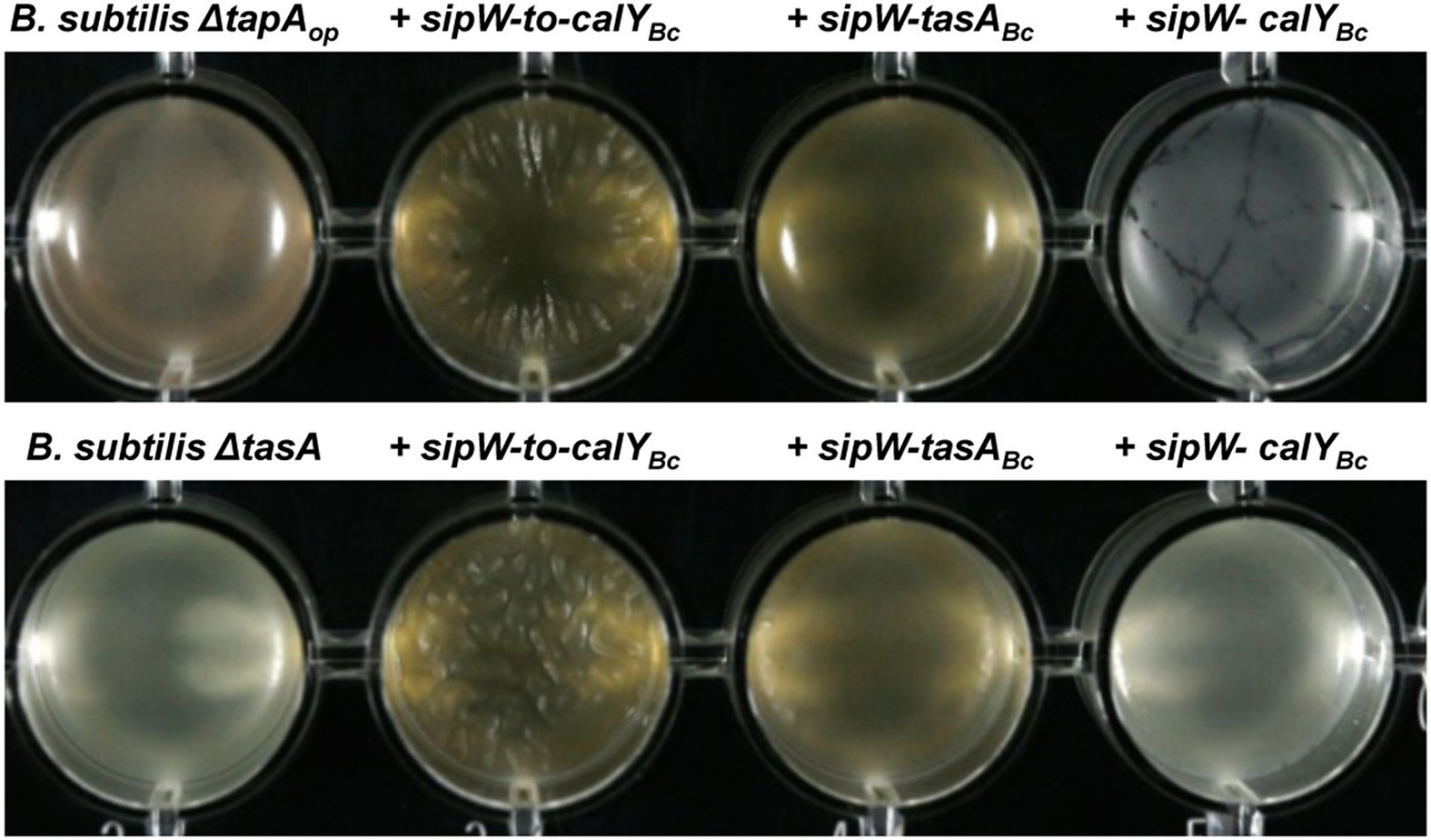
**Heterologous expression of *B. cereus sipW-to-tasA* or *sipW-tasA* in *B. subtilis* rescues pellicle formation**. *B. cereus* alleles were ectopically integrated at the *lacA* or *amyE* locus of *B. subtilis* mutants lacking the entire *tapA* operon (*B. subtilis ΔtapA_op_*) or lacking *tasA* alone (*B. subtilis ΔtasA*); expression of these alleles was driven by an IPTG-inducible promoter. Biofilm experiments were performed in static cultures in MSgg broth and induced with 1 mM IPTG in 24-well plates. Top view of pellicles were photographed after 48 h of incubation in MSgg broth at 30°C.

**Figure 6:**
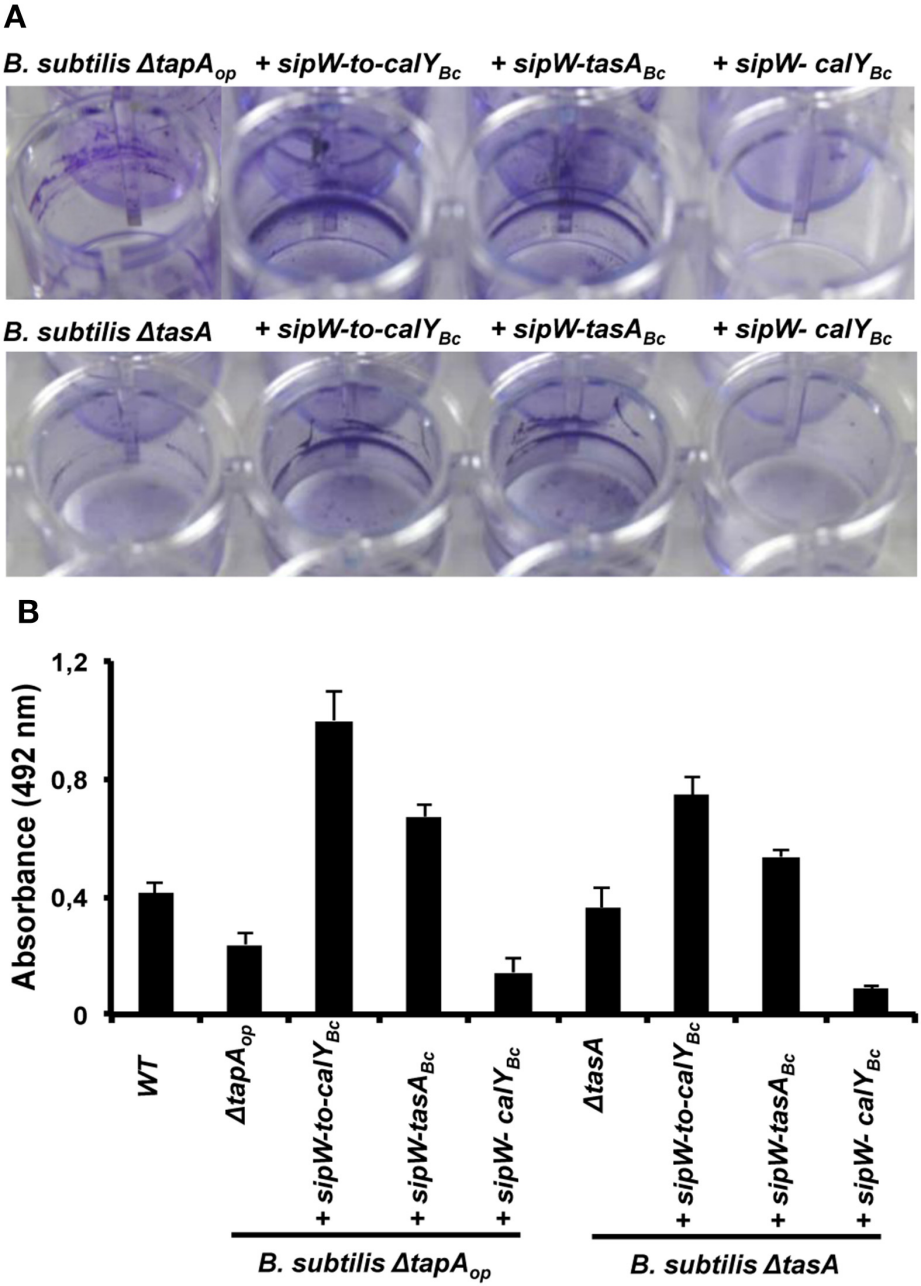
**Heterologous expression of *B. cereus sipW-to-tasA* or *sipW-tasA* in *B. subtilis* enhances adhesion to abiotic surfaces**. *B. cereus* alleles were ectopically integrated at the *lacA* or *amyE* locus of *B. subtilis* mutants lacking the entire *tapA* operon (*B. subtilis ΔtapA_op_*) or lacking *tasA* alone (*B. subtilis ΔtasA*); expression of these alleles was driven by an IPTG-inducible promoter. Biofilm experiments were performed in static cultures in MSgg broth and induced with 1 mM IPTG in 24-well plates. **(A)** Adhesion to abiotic surfaces was examined by staining the cultures with Crystal Violet, and pictures were obtained after 48 h of incubation at 30°C. **(B)** Quantification of the amount of crystal violet retained in bacterial biomass. Data are the average and standard deviation from four independent experiments consisting of six internal replicates each.

Our results suggested that the *sipW-tasA* and *calY* are involved in *B. cereus* biofilm formation and that TasA is important for assembly of pellicles in the air-liquid interphase. Therefore, we proposed that *B. cereus* TasA might functionally replace *B. subtilis* TasA. *B. subtilis* forms wrinkly pellicles in the air-liquid interphase; this property facilitates identification of mutants that disrupt assembly of the normal architecture. In *B. subtilis*, deletion of TasA leads to a defect in assembling wrinkly pellicles (Branda et al., 2006). Therefore, we performed heterologous expression of the respective *B. cereus* loci (referred to as *allele_Bc_*) in *B. subtilis* and examined the pellicle phenotype (Figure 5) and the adhesion to abiotic surfaces (Figure 6). The constructs were ectopically integrated at the *amyE* or *lacA* locus of *B. subtilis*, and their expression was driven by an IPTG-inducible promoter to bypass any potential regulatory processes associated with their native promoters.

To examine the role of TasA in pellicle formation, we complemented a *B. subtilis* mutant lacking the *tapA* operon (Δ*tapA_op_*) with the *B. cereus sipW*-to-*calY* chromosomal region (*sipW-to-calY_Bc_*). Our RT-PCR analysis indicated that *calY* is a monocistronic gene independent of *sipW-tasA* (Figure 1B), thus in this construct *(P_hyperspank_-sipW-to-calY_Bc_*) the expression of *calY* must rely under the control of its own promoter. As expected, this *B. cereus* construct rescued the formation of wrinkly pellicles, which resembled wild-type *B. subtilis* pellicles (Figure 5); furthermore, this construct notably enhanced the adhesion to the well surfaces in crystal violet assays (Figures 6A,B, top row). Therefore, the *B. cereus sipW*-to-*calY* region is involved in pellicle formation. Next, we examined the specific roles of *tasA* and *calY* in pellicle formation. Expression of either *tasA* or *calY* failed to restore pellicle formation in the *B. subtilis tasA* mutant strain (Δ*tasA)* (Figure S1, top and middle rows). We then tested whether these loci must be expressed with the cognate *sipW* gene of *B. cereus*. Expression of *sipW-tasA* in the *B. subtilis* Δ*tasA* mutant strain rescued pellicle formation (Figure 5, bottom row) and adhesion to abiotic surfaces, although less efficiently than that complementation with *sipW-to-calY* (Figures 6A,B bottom row). Notably, expression of the *sipW-calY* construct failed to restore pellicle formation (Figure 5, bottom row) or bind to abiotic surfaces (Figures 6A,B). Because the *B. subtilis ΔtasA* mutant strain encodes the *tapA* gene, which is required to form pellicles in *B. subtilis* (Romero et al., 2011) we expressed *sipW-tasA* or *sipW-calY* in a *B. subtilis* strain lacking the entire operon Δ*tapA_op_*; this strategy eliminated expression of TapA, a protein with no ortholog in *B. cereus* but retained the native *sipW* gene. We observed that *sipW-calY* did not restore the wild-type phenotype (Figure 5, top row; Figures 6A,B top row), and *sipW-tasA* complementation resulted in the formation of pellicles but not wrinkles (Figure 5, top row) and restored adhesion to abiotic surfaces (Figures 6A,B top row).

These results suggested that *B. subtilis tapA* might affect pellicle formation in the *B. subtilis ΔtasA* mutant complemented with *B. cereus sipW-tasA*. Therefore, we examined pellicle formation in a *B. subtilis ΔtapA* mutant strain complemented with the following *B. cereus* regions: *sipW-to-calY*, *sipW-tasA*, or *sipW-calY* (Figure S2, top and middle rows). We observed that expression of *sipW-to-calY* was required to restore formation of wrinkly pellicles and adhesion to abiotic surfaces. Expression of *sipW-tasA* partially rescued the mutant phenotype, but *sipW-calY* failed to restore any of these phenotypes. Together, these observations confirmed the intrinsic ability of the protein products of the *B. cereus sipW-to-calY* region to facilitate the formation of wrinkly pellicles in the surrogate host *B. subtilis*. Additionally, TasA and CalY might have complementary roles in this phenotype, but the function of TasA is predominant.

### *B. cereus* TasA mimics the formation of amyloid-like fibers in *B. subtilis* biofilms

Two observations indicated that *B. cereus* TasA has amyloid-like properties: (i) *B. cereus* cells contained polymerized fibers (Figure 3), and (ii) the thin pellicles in *B. cereus* cells expressing TasA were stained with Congo Red (Figure 4). Based on these observations, we proposed that *B. cereus* TasA would display similar behavior and aggregate into amyloid-like fibers when expressed in the surrogate host *B. subtilis*.

**Figure 7:**
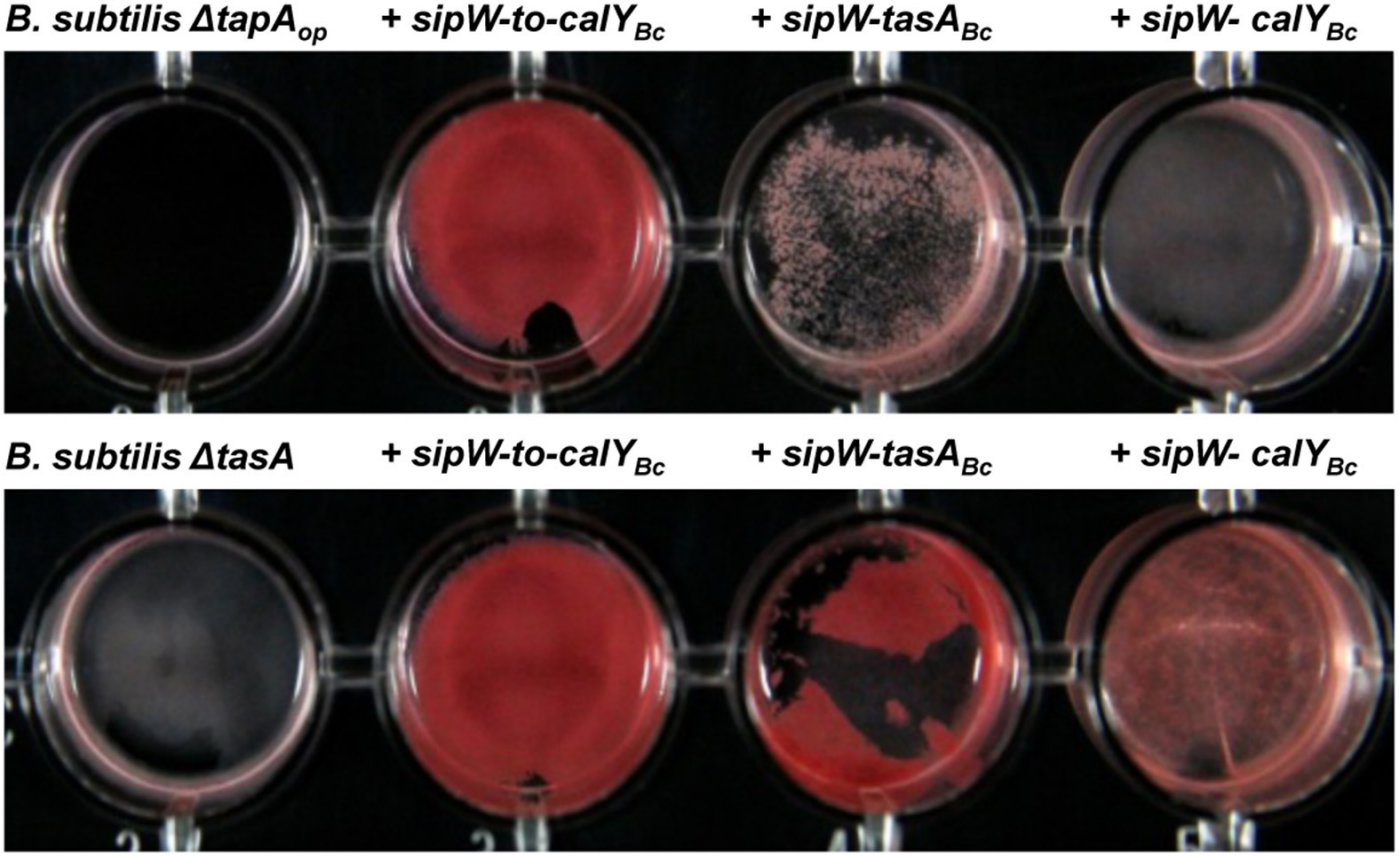
**Pellicles of *B. subtilis* complemented with *sipW-to-tasA* or *sipW-tasA of B. cereus* stained with specific amyloid dye Congo Red**. *B. cereus* alleles were ectopically integrated at the *lacA* or *amyE* locus of *B. subtilis* mutants lacking the entire *tapA* operon (*B. subtilis ΔtapA_op_*) or lacking *tasA* alone (*B. subtilis ΔtasA*); expression of these alleles was driven by an IPTG-inducible promoter. Biofilm experiments were performed in static cultures in Ty-Congo Red (20 μg/ml)-Coomassie Blue (20 μg/ml) broth and induced with 1 mM IPTG in 24-well plates. Top-view pictures of pellicles after 24 h of growth at 30°C with no agitation.

To test this hypothesis, we grew biofilms from mutant *B. subtilis* cells and cells complemented with different *B. cereus* loci; the cells were grown in TY medium supplemented with the amyloid-specific dye Congo Red (Figure 7). As expected, the *B. subtilis ΔtapA_op_* and Δ*tasA* mutants did not bind this dye. Staining with Congo Red was stronger in pellicles of *B. subtilis ΔtapA_op_* mutant cells complemented with the entire *sipW-to-calY* region of *B. cereus* (Figure 7, top row); a strain that, as described above, formed wrinkly pellicles (Figure 5). The pellicles of *B. subtilis ΔtapA_op_* strains expressing either *sipW-tasA* or *sipW-calY* were stained at low levels (Figure 7, top row). Notably, complementation of the *B. subtilis ΔtasA* mutant strain with the *sipW-tasA* region or *sipW-to-calY* resulted in similar levels of Congo Red staining; however, complementation with the *sipW-calY* construct resulted in weaker staining (Figure 7, bottom row). These data were consistent with the results obtained on pellicle formation and suggested that *B. subtilis tapA* influences cellular staining properties. However, when these experiments were performed using a *B. subtilis ΔtapA* mutant strain, complementation with the entire *B. cereus sipW-to-calY* or *sipW-tasA* but not *sipW-calY* restored Congo Red binding (Figure S2, bottom row).

**Figure 8:**
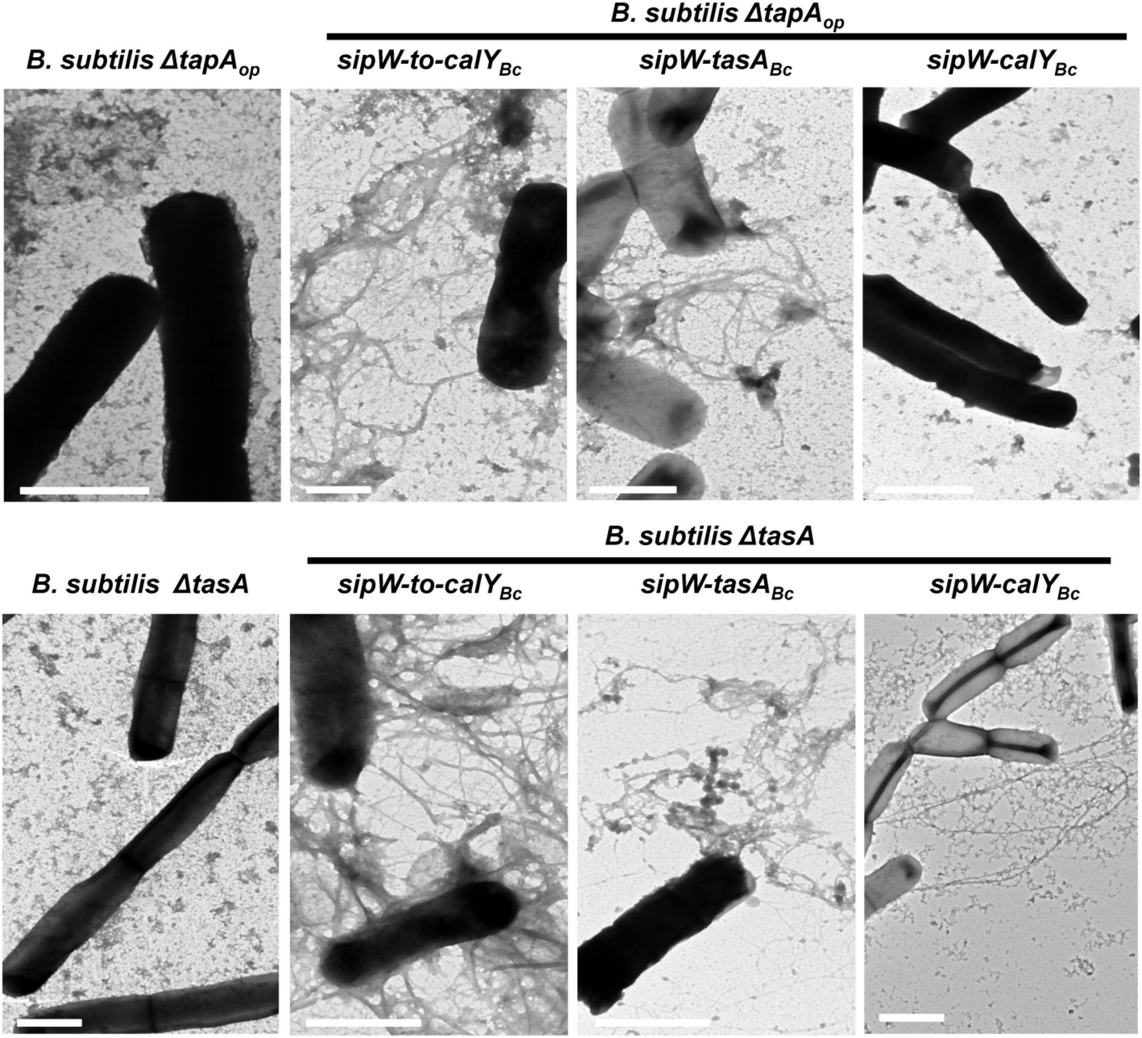
**Expression of *B. cereus sipW-tasA* leads to fiber formation in *B. subtilis***. *B. cereus* alleles were ectopically integrated at the *lacA* or *amyE* locus of *B. subtilis* mutants lacking the entire *tapA* operon (*B. subtilis ΔtapA_op_*) or lacking *tasA* alone (*B. subtilis ΔtasA*), and their expression was driven by an IPTG-inducible promoter. The strains were grown in MSgg broth and induced with 1 mM IPTG without shaking at 30°C. Samples were collected after 24 h, contrasted using uranyl acetate and then analyzed using transmission electron microscopy. Scale bars = 1 μm.

To further examine the amyloid nature of *B. cereus* TasA and CalY, we studied fibrillation of TasA and CalY on the *B. subtilis* cell surfaces (Figure 8). Transmission electron microscopy analysis revealed that *B. subtilis ΔtasA_op_* and Δ*tasA* mutant cells contained no fibers. Consistent with other experiments *B. subtilis ΔtasA_op_* cells complemented with the *B. cereus sipW-to-calY* or *sipW-tasA* loci appeared decorated with several fibers (Figure 8, top row). However, complementation with *sipW-calY*, which did not restore Congo Red binding, also failed to promote fiber formation (Figure 8, top row). Expression of the entire *B. cereus sipW-to-calY* region or *sipW-tasA* resulted in the formation of abundant fibers on the surfaces of *B. subtilis ΔtasA* cells; however, expression of the *sipW-calY* promoted less abundant and thinner fibrils formation (Figure 8, bottom row). Finally in *B. subtilis ΔtapA* mutant; fibers were formed in Δ*tapA* cells complemented with the entire region or with *sipW-tasA* but not with *sipW-calY* (Figure S3). These results indicated that (i) the entire region of *B. cereus* drives the formation of fibers with amyloid properties similar to the *tapA_op_* of *B. subtilis* but does not require a *tapA* ortholog, (ii) *B. cereus* TasA and a at lesser extent CalY polymerizes to form fibers similar to *B. subtilis* TasA, and (iii) CalY complements the function of TasA in the formation of pellicles and fibers.

## Discussion

The bacterium *Bacillus cereus* is widely distributed in nature, and several species within this group inhabit soils, colonize arthropod guts or are pathogenic to humans (Bottone, 2010). The intrinsic factors contributing to this versatile ecological distribution include spore and biofilm formation (Auger et al., 2009; Elhariry, 2011). Spores are highly resistant to environmental stresses and are extremely adhesive, facilitating attachment to abiotic and biotic surfaces (Ball et al., 2008; Shaheen et al., 2010). Biofilms are considered to promote adhesion and protect cells from antimicrobials and other external insults and are thus difficult to eradicate (Flemming and Wingender, 2010). In this study, we examined the role of a specific genomic region in *B. cereus* biofilm formation.

Studies on biofilms of the phylogenetically related organism *B. subtilis* have elucidated both the genetic circuits that govern the biofilm developmental program and the structural components that facilitate assembly of the extracellular matrix (Romero, 2013; Vlamakis et al., 2013). The important matrix components of *B. subtilis* biofilms include exopolysaccharides, the hydrophobin BlsA and the amyloid-like protein TasA (Branda et al., 2004; Romero et al., 2010; Ostrowski et al., 2011). TasA, TapA and the signal peptidase SipW are especially important for correct assembly of the extracellular matrix. The studied *B. cereus* genomic region contains two TasA orthologs, TasA and CalY, and an ortholog of the signal peptidase SipW. However, this region lacks the accessory protein TapA. The fact that the expression of *sipW*, *tasA* and *calY* of *B. cereus* are under control of the biofilm master regulator SinR (Fagerlund et al., 2014) led to think in their implication in biofilm formation, as the *tapA_op_* in *B. subtilis* (Chu et al., 2006), and our data are supportive of this hypothesis (Figure 2). The divergent patterns of biofilm formation in *tasA* and *calY* mutants suggested that these proteins function in different stages of biofilm formation: CalY might be more important for initial attachment, and TasA might be required for further maturation. Indeed, pioneer studies demonstrated that CalY could be purified from *B. cereus* cells at mi-log phase of growth (Fricke et al., 2001). In the other hand, this observation is not unprecedented; the interplay of diverse factors in biofilm formation can be observed in other bacteria species. The Gram-negative bacterium *Pseudomonas putida* contains two protein adhesins, LapF and LapA, which are essential for the initiation and maturation of biofilms, respectively (Martinez-Gil et al., 2010). We further propose that CalY and TasA are required for cell-to-cell and cell-to-abiotic surface interactions. However, crystal violet staining and heterologous expression analyses in *B. subtilis* cells revealed that TasA might be more important for the interaction to abiotic surfaces (Figure 6).

The *B. cereus tasA* mutant cells form an early biomass ring that is loosely bound to abiotic surfaces; therefore, other extracellular matrix components might be over-expressed. Two possible candidates are an exopolysaccharide or CalY. In *B. subtilis*, the absence of TasA increases the expression of the exopolysaccharide by an unknown regulatory pathway (Vlamakis et al., 2008), and a similar imbalance in the expression of components of the extracellular matrix has been reported in *P. putida* mutants lacking its large adhesin proteins (Martinez-Gil et al., 2013). Consistent with this observation, pellicles of a *B. subtilis ΔtasA* mutant were easy to disrupt and fluid; expression *of B. cereus sipW*-*tasA* suppressed these defects, and the pellicles resembled those of wild-type *B. subtilis* cells (Figure 5), indicating that EPS levels might be restored. The genome of *B. cereus* contains a region (*BC_5267* to *BC_5278*) that highly resembles the operon dedicated to the synthesis of EPS in *B. subtilis*, but contrary to this bacteria species, the loci of *B. cereus* do not appear to be part of the *sinR* regulon (Fagerlund et al., 2014). Whether these or additional unknown factors of *B. cereus* are involved in biofilm formation needs to be clarified.

To build the extracellular matrix, *B. subtilis* TasA forms resistant fibers with amyloid properties. This process requires an accessory protein TapA, which contributes to the initiation and growth of TasA fibers (Romero et al., 2011, 2014). Our data from mutagenesis in *B. cereus* and heterologous expression in *B. subtilis* indicate that the *B. cereus sipW-to-calY* region contains all elements required for fiber assembly (Figures 3, 8). We propose that as previously described in *B. subtilis*, SipW functions as a signal peptidase that processes TasA and CalY to their mature forms for secretion (Tjalsma et al., 1998; Stover and Driks, 1999a,b). The rationale for this hypothesis is as follows: first, both proteins contain signal peptides with a canonical sequence that is a substrate for SipW proteolytic activity (Tjalsma et al., 1998; Terra et al., 2012); second, a *sipW* mutant is completely defective for biofilm formation (Figure 2); third, *B. cereus* TasA and CalY are not functional in *B. subtilis* unless they are co-expressed with the cognate SipW protein (Figure 5 and Figure S1). Our data suggest that *B. cereus* TasA is the more important for fiber formation than the other *B. subtilis*-TasA ortholog CalY. Abundant TasA fibers are present in wild-type and *calY* mutant *B. cereus* cells (Figure 3). Furthermore, in heterologous expression experiments, *B. subtilis* mutants lacking *tapA* formed fibers using *B. cereus* TasA (Figure 8). CalY has 62% identity with *B. cereus* TasA; CalY assembled into thin fibrils in *B. cereus* but failed to form fibers in *B. subtilis* unless TapA was present. One interpretation is that TapA of *B. subtilis* is able to cross seed the assembly of CalY fibers, a phenomenon recently reported in the assembly of the amyloid-like fiber Curli among *Eschirichia coli* and *Salmonella typhimurium* (Zhou et al., 2012).

Besides all our observations, we do not exclude the possibility that CalY has amyloid-like properties. Previous studies have shown that CalY is unusually resistant to SDS and heat treatments, and display high aggregative properties in organic solvent (Fricke et al., 2001); features associated with but not exclusive to amyloid proteins (Greenwald and Riek, 2010). The high sequence identity of CalY and TasA leads to the following mutually exclusive models: (i) CalY and TasA cooperate to assemble robust and stable fibers with amyloid properties including binding Congo Red, as described among CsgA and CsgB in assembly of Curli in *E. coli* (Shu et al., 2012); (ii) TasA and CalY form fibers independently, but these two types of fibers are important for biofilm assembly during different environmental conditions. An example of the diversification of amyloid-like proteins is the Gram-positive bacterium *Streptomyces coelicolor*, which has up to eight different chaplin proteins with a propensity to assemble amyloid-like fibers; this suggests the existence of significant plasticity to ensure the completion of complex developmental programs (Di Berardo et al., 2008; Sawyer et al., 2011). Further biochemical and morphological analyses of purified TasA and CalY are required to elucidate their amyloid properties.

In summary, we identified a specific *B. cereus* genomic region, which contains two independent genetic factors, the two-gene operon *sipW-tasA* and the gene *calY*, which are both necessary for *B. cereus* biofilm formation. Directed mutagenesis in *B. cereus* and heterologous expression of the *B. cereus* alleles in *B. subtilis* revealed that TasA and, to a lesser extent, CalY have the intrinsic ability to polymerize and form fibers that are microscopically similar to the TasA amyloid-like fibers of *B. subtilis*. Finally, the Congo Red-binding ability of pellicles in TasA-expressing *B. cereus* cells and in *B. subtilis* cells complemented with *sipW-tasA* and CalY or only *sipW-tasA* point toward the amyloid nature of fibers formed by TasA.

### Conflict of interest statement

The authors declare that the research was conducted in the absence of any commercial or financial relationships that could be construed as a potential conflict of interest.
