# Supplementary material for: A genomic region involved in the formation of adhesin fibers in Bacillus cereus biofilms
Source: Front Microbiol. 2015 Jan 13;5:745. doi: 10.3389/fmicb.2014.00745 (PMC4292775; doi:10.3389/fmicb.2014.00745)
Supplement: Supplementary file 1 [file Presentation1.PPT]

## Slide 1
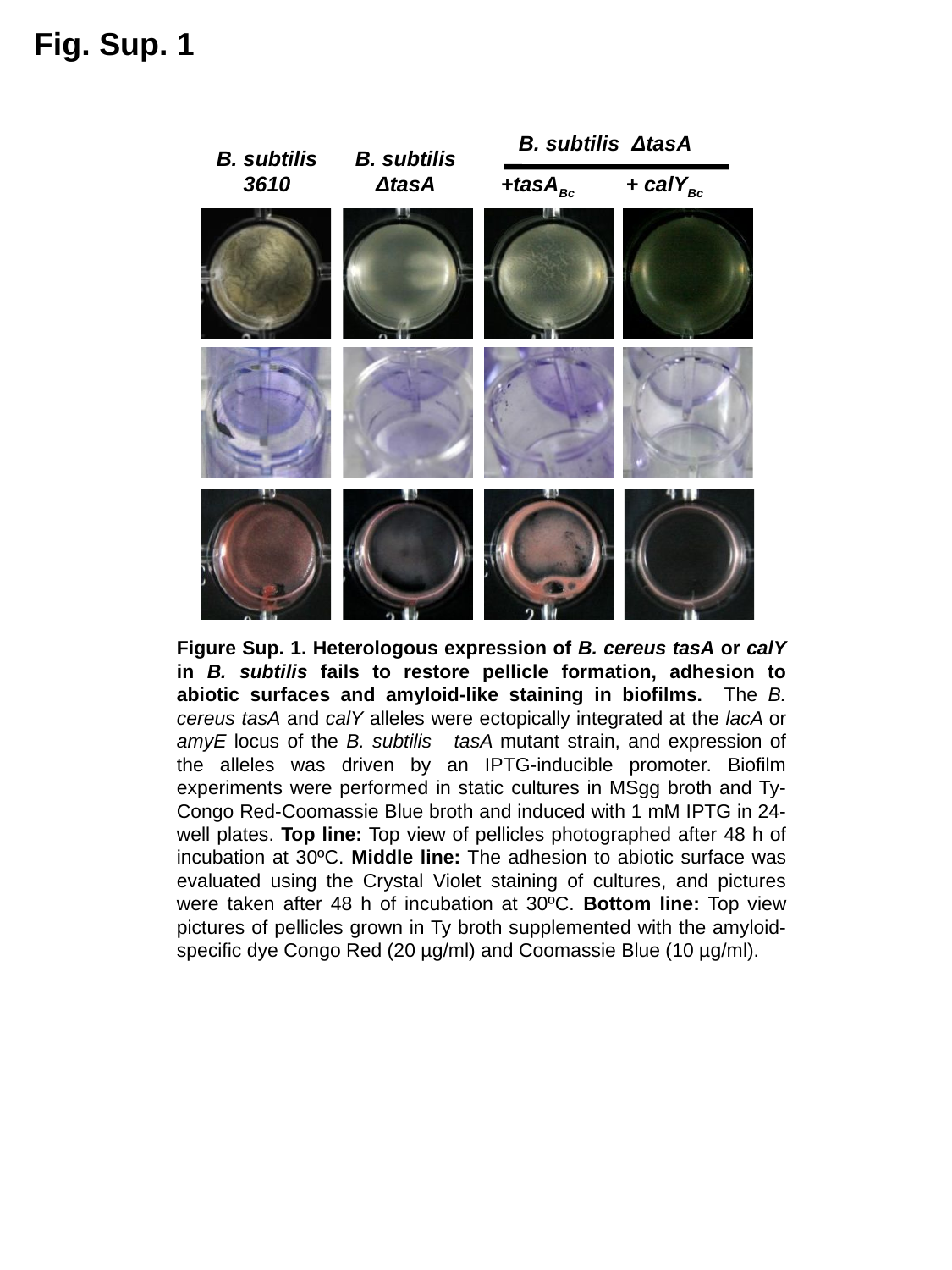

Fig. Sup. 1
B. subtilis ΔtasA
B. subtilis 3610
B. subtilis ΔtasA
+tasABc
+ calYBc
Figure Sup. 1. Heterologous expression of B. cereus tasA or calY in B. subtilis fails to restore pellicle formation, adhesion to abiotic surfaces and amyloid-like staining in biofilms. The B. cereus tasA and calY alleles were ectopically integrated at the lacA or amyE locus of the B. subtilis ΔtasA mutant strain, and expression of the alleles was driven by an IPTG-inducible promoter. Biofilm experiments were performed in static cultures in MSgg broth and Ty-Congo Red-Coomassie Blue broth and induced with 1 mM IPTG in 24-well plates. Top line: Top view of pellicles photographed after 48 h of incubation at 30ºC. Middle line: The adhesion to abiotic surface was evaluated using the Crystal Violet staining of cultures, and pictures were taken after 48 h of incubation at 30ºC. Bottom line: Top view pictures of pellicles grown in Ty broth supplemented with the amyloid-specific dye Congo Red (20 µg/ml) and Coomassie Blue (10 µg/ml).

## Slide 2
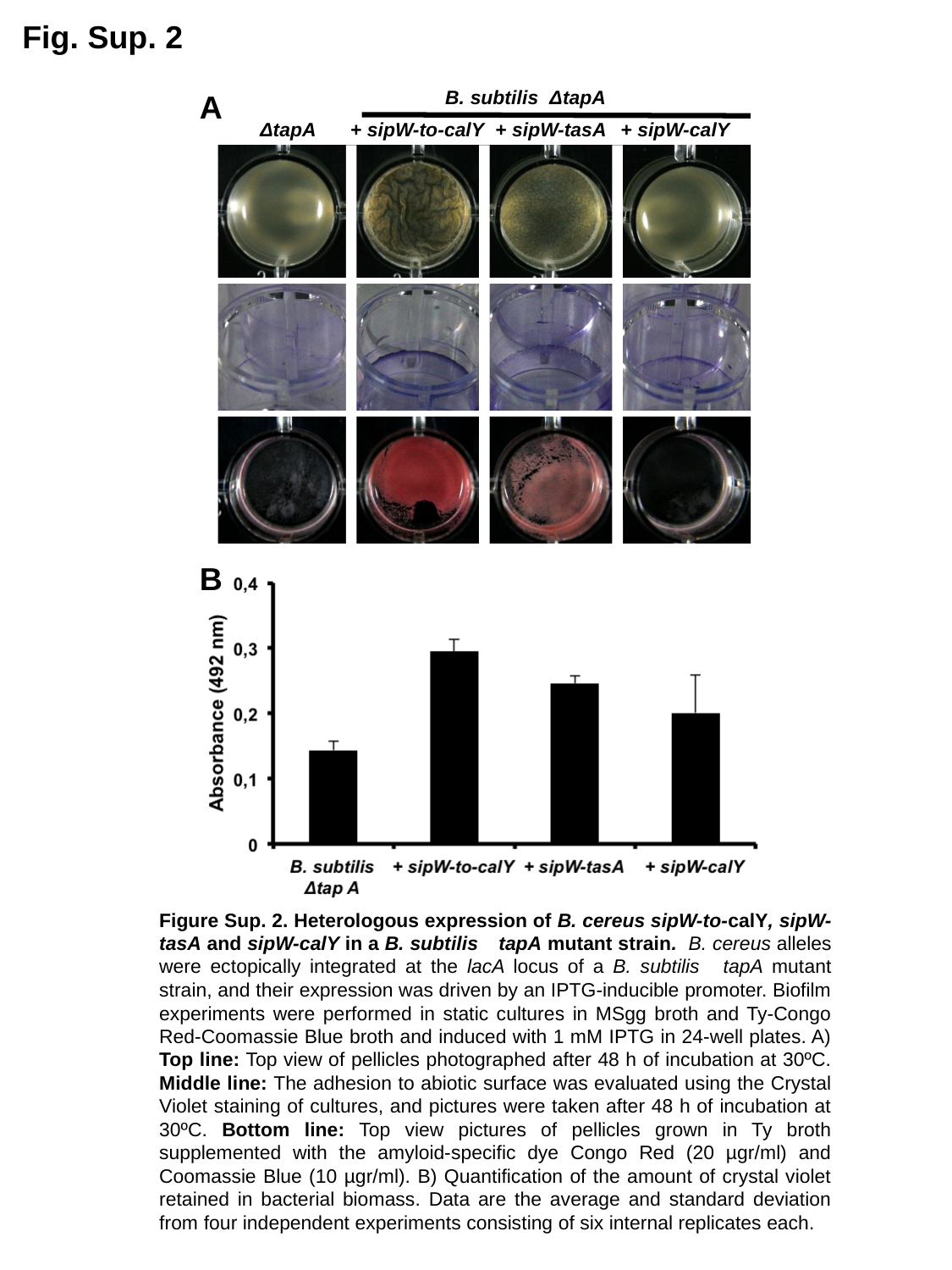

Fig. Sup. 2
A
B. subtilis ΔtapA
ΔtapA
+ sipW-to-calY
+ sipW-tasA
+ sipW-calY
B
Figure Sup. 2. Heterologous expression of B. cereus sipW-to-calY, sipW-tasA and sipW-calY in a B. subtilis ΔtapA mutant strain. B. cereus alleles were ectopically integrated at the lacA locus of a B. subtilis ΔtapA mutant strain, and their expression was driven by an IPTG-inducible promoter. Biofilm experiments were performed in static cultures in MSgg broth and Ty-Congo Red-Coomassie Blue broth and induced with 1 mM IPTG in 24-well plates. A) Top line: Top view of pellicles photographed after 48 h of incubation at 30ºC. Middle line: The adhesion to abiotic surface was evaluated using the Crystal Violet staining of cultures, and pictures were taken after 48 h of incubation at 30ºC. Bottom line: Top view pictures of pellicles grown in Ty broth supplemented with the amyloid-specific dye Congo Red (20 µgr/ml) and Coomassie Blue (10 µgr/ml). B) Quantification of the amount of crystal violet retained in bacterial biomass. Data are the average and standard deviation from four independent experiments consisting of six internal replicates each.

## Slide 3
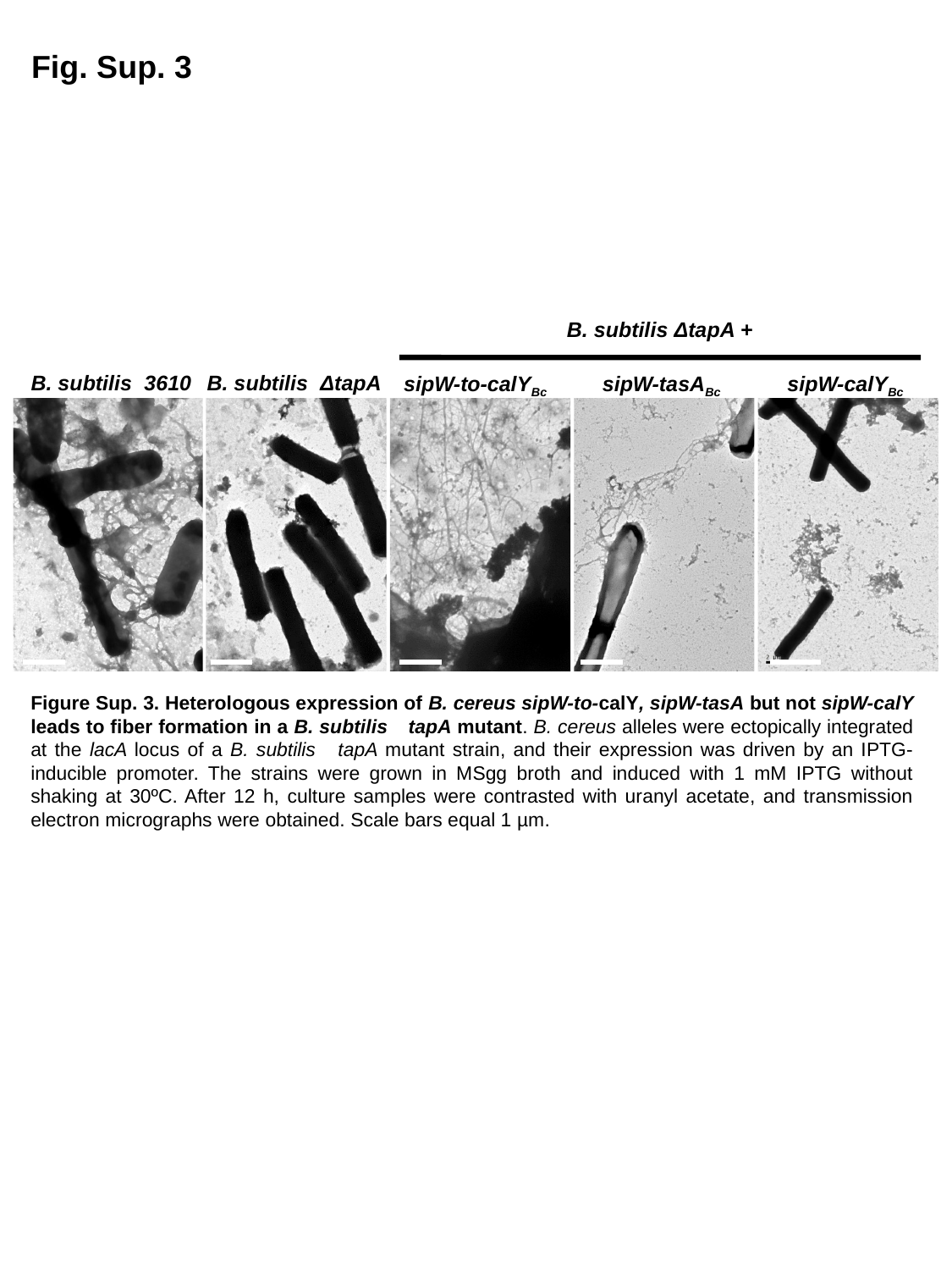

Fig. Sup. 3
B. subtilis ΔtapA +
B. subtilis 3610
B. subtilis ΔtapA
sipW-to-calYBc
sipW-tasABc
sipW-calYBc
Figure Sup. 3. Heterologous expression of B. cereus sipW-to-calY, sipW-tasA but not sipW-calY leads to fiber formation in a B. subtilis ΔtapA mutant. B. cereus alleles were ectopically integrated at the lacA locus of a B. subtilis ΔtapA mutant strain, and their expression was driven by an IPTG-inducible promoter. The strains were grown in MSgg broth and induced with 1 mM IPTG without shaking at 30ºC. After 12 h, culture samples were contrasted with uranyl acetate, and transmission electron micrographs were obtained. Scale bars equal 1 µm.
