# Supplementary material for: A genomic region involved in the formation of adhesin fibers in Bacillus cereus biofilms
Source: Front Microbiol. 2015 Jan 13;5:745. doi: 10.3389/fmicb.2014.00745 (PMC4292775; doi:10.3389/fmicb.2014.00745)
Supplement: Supplementary file 2 [file DataSheet1.PDF]

Table Sup. 1. List of primers used in this study

**Mutations of *B. cereus***		
Primers to mutate *sipW*	BamHI_SipW.F.up	AAAAGGATCCCCGATTCGTGAGGGTTGATG
	sipW-R_Up_mls	GAGGGTTGCCAGAGTTAAAGGATCCCATATTAATTTCATCGTCTCTCTCCCTCTCCG
	sipW-F_Down_mls	CGATTATGTCTTTTGCGCAGTCGGCGATAAAAAAGTAGAACAATCCGTCTAGTT
	NcoI_SipW.R.down	AAAACCATGGCAATGCTGCTGATGCAACTC
Primers to mutate *tasA*	BamHI_TasA.F.up	AAAAGGATCCCCATAACATTGCGTGCTGAG
	TasAR-up_Km	CCTATCACCTCAAATGGTTCGCTGTAAAGTCATGTCCATATCCCCTTAATA
	TasAF-D_Km	CGAGCGCCTACGAGGAATTTGTATCGCAATTAAATTGGACGTTTGATGCACAACAA
	NcoI_TasA.R.down	AAAACCATGGCTAACCTAGACTCCGAATGG
Primers to mutate *calY*	BamHI_CalY.F.up	AAAAGGATCCGGTGCTATTCGTAGCATTGAC
	CalR-up_mls	GAGGGTTGCCAGAGTTAAAGGATCACTCACAATCAATTCCCCCTAGCTTTT
	Long_CalF-D_mls	CGATTATGTCTTTTGCGCAGTCGGCCATTGAATTTAGAATGGACATTC
	NcoI_CalY.R.down	AAAACCATGGTGTAGTCGAACGCTGCCATC
Primers to mutate *sipW-to-calY*	BamHI_SipW.F.up	AAAAGGATCCCCGATTCGTGAGGGTTGATG
	sipW-R_Up_mls	GAGGGTTGCCAGAGTTAAAGGATCCCATATTAATTTCATCGTCTCTCTCCCTCTCCG
	Long_CalF-D_mls	CGATTATGTCTTTTGCGCAGTCGGCCATTGAATTTAGAATGGACATTC
	NcoI_CalY.R.down	AAAACCATGGTGTAGTCGAACGCTGCCATC
**RT-PCR**		
	SipW_F	AGATAATTAGCAACGCGATCTC
	SipW_R	AGAAATAGCGGAATAACCAAGC
	TasA_F	AGCAGCTTTAGTTGGTGGAG
	TasA_R	GTAACTTATCGCCTTGGAATTG
	BC1280_F	GTAGAAGCGACGCTTTCTAC
	BC1280_R	TGTCCTTCCGCTTCAATTGC
	CalY_F	AGGTATGGGAGTTGCATCAG
	CalY_R	CAGCTTCTTGGTTGGCATTG
	prev-SipW_Fw	GCATTGGCGGTAAGACAGGG
	prev-SipW_R	GATCGCGTTGCTAATTATCTTCC
	SipW-TasA_Fw	TTGGTGCTATTCGTAGCATTGAC
	SipW-TasA_R	CAGATTTACCTGCTGAAATACC
	TasA-1280_Fw	ATCGGCTTGGTTCTGGGATG
	TasA-1280_R	TTTCTCACGCCCTTGTTGCC
	1280-CalY_Fw	GGAACAGAAGAAAGTAGAAGAAC
	1280-CalY_R	GCTGATGCAACTCCCATACC
	CalY-next_Fw	GGTGGATTAGCAGCTGGTACAGAG
	CalY-next_R	AGACAGACGCACTGGATCAAG
**Heterologous expression in**		

B. subtilis

Primers to express *sipW-calY*	SipWF_salI	AAAAAAAGTCGACAACGGAGAGGGAGAGAGACGATGAAATTA
	SipW-Rev	CGAACTAGACGGATTGTTCTAC
	SipWtail-CalYFw	AAAGTAGAACAATCCGTCTAGTTCGAGAACGCATATTAGCTGAGC
	BC1281R_sphI	AAAAAAAGCATGCTTATTATTTTTCTTCCCCAGCTTCTTGGTT
Primers to express *sipW-tasA*	SipWF_salI	AAAAAAAGTCGACAACGGAGAGGGAGAGAGACGATGAAATTA
	TasAR_sphI	AAAAAAAGCATGCTTTTTCTATTTTTCTTCACCTGCTGTTTG TTG
Primers to express *tasA*	TasAR_sphI	AAAAAAAGCATGCTTTTTCTATTTTTCTTCACCTGCTGTTTGTTG
	TasAF_salI	AAAAAAAGTCGACAAATATATATTAAGGGGATATGGACATGACTTTA
Primers to express *calY*	BC1281_salI	AAAAAAAGTCGACAAAAAGCTAGGGGGAATTGATTGTGAGTCTG
	BC1281R_sphI	AAAAAAAGCATGCTTATTATTTTTCTTCCCCAGCTTCTTGGTT
Primers to expresss *sipW-to-calY*	SipWF_salI	AAAAAAA GTCGACAACGGAGAGGGAGAGAGACGATGAAATTA
	BC1281R_sphI	AAAAAAAGCATGCTTATTATTTTTCTTCCCCAGCTTCTTGGTT
